# Analysis of Factors Influencing the Grading of Condition Severity and Zoning Management in an Emergency Triage System

**Published:** 2017-01

**Authors:** Yongxia SUN, Xiuping WANG, Huiping XUE, Xiuzhen LI

**Affiliations:** 1. Emergency Dept., Binzhou Central Hospital of Shandong Province, Binzhou, PR China; 2. ICU, Binzhou Central Hospital of Shandong Province, Binzhou, PR China

**Keywords:** Patient, Emergency triage, Influencing factors, Hospital

## Abstract

**Background:**

To identify and analyze factors that influence the grading and zoning management model of emergency triage as a first step in the improvement of the system.

**Methods:**

Questionnaires were used to extract data from clinical histories of 286 patients who attended the Emergency Department of Binzhou Central Hospital (Binzhou City, Shandong Province, China) from September to November of 2015. Through logistic regression analysis, influencing factors for unreasonable (≥ 2h) emergency department retention times were identified.

**Results:**

Analysis of general characteristics of patients including gender, method of payment or assigned medical department did not result in any statistically significant differences between patients with a time to discharge longer than 2 h and those with a shorter time to discharge (P > 0.05). Older age, higher income, lower or higher education degree, admission time from 17:00–7:59, and lack of understanding of zoning management and of condition severity grading resulted in a retention time greater than or equal to 2 hours (P < 0.05). According to the Logistic regression model: age, income level, education degree, admission time, degree of understanding of zoning management and condition severity grading were all independent risk factors affecting the time to discharge from the emergency department (P <0.05).

**Conclusion:**

Countermeasures need to be developed in order to minimize the influence of patients’ factors and promote reasonable average retention times lower than 2 hours in the emergency department.

## Introduction

With the improvement of living standards and the acceleration of the tempo of social life, the people’s demand for health attention is also increasing. The emergency departments of comprehensive hospitals can be overcrowded with patients, and the circulation time of outpatients can be very unreasonable, causing a deficit of valuable resources that would better be employed during veritable emergencies (1). This situation leads to a wasteful use of medical resources in hospitals that eventually run out of sufficient supplies to effectively meet the medical needs of patients, and leads to a decline in the quality of emergency medical care, an increase of preventable adverse events, prolonged hospitalization times, increase of medical expenses and an increase in the hospital mortality rate (a serious manifestation of medical system dysfunction) (2). In order to improve these situations, hospitals need to have in place a reasonable regulation of the inflow and outflow routes and an effective emergency triage system.

China’s Ministry of Health issued guidelines and standards on the emergency triage system back in 2011 and again in 2012. The zoning management model, evaluates the patients’ conditions giving scores of 1–4 grades according to the severity and the need to occupy emergency medical resources. The emergency treatment area is divided into red, yellow and green parts according to the space layout, patients on grades 1 and 2 are arranged into the red zone to receive immediate treatment, patients on grade 3 go to a yellow intermediate zone and patients on grade 4 await treatment on a green zone. This zoning method is supposed to classify effectively patients (3–4).

This study aimed at evaluating the experience of 286 emergency patients and their relatives through analysis of questionnaire responses, and then to identify risk factors influencing the effects of zoning management.

## Materials and Methods

Overall, 286 patients treated in the Emergency Department of Binzhou Central Hospital (Binzhou City, Shandong Province, China) from December to November 2015 and their families were randomly selected; one member of each family was chosen to answer a questionnaire. Totally, 144 patients were male and 142 were female; their ages ranged from 20–65 yr (41.7±8.5 yr on average); 65 cases had an education level below junior high school, 159 had a high school and polytechnic school level, and 62 had a level above junior college. From 300 questionnaires handed out initially, 286 were handed in and were used for the study. The effective rate of the initial study recruitment was of 95.33%.

This study was approved by the Ethics Committee of Binzhou Central Hospital. Signed written informed consents were obtained from all participants before the study.

### Research methods

The questionnaire employed was specially designed for this study; its content included various sections as follows: A basic personal information section with the patients’ gender, age, education level, career, residential address, income level, payment method, and other related data. A section to measure how well the patient informed was: understanding of the grading and zoning system, understanding of the reasons for treatment and for the assignment to a particular medical department, etc., in addition to a section comprising other aspects such as treatment difficulties and satisfaction, etc. The questionnaire was handed out to patients and their families by specially trained investigators who explained how to fill correctly it, and then waited for the questionnaires to complete on site.

### Statistical analysis

The Epidata 3.1 software was used for data input; after data exporting, Excel 2010 was used for data arrangement, and SPSS 19.0 (Chicago, IL, USA) was used for statistical analysis. Measurement data were expressed by the mean ± standard deviation and tested by the t-test. Count data were expressed by number of cases or a percentage and tested by χ^2^. The correlation analysis was done using the Spearman correlation, and a Logistic regression analysis model was used to determine influencing factors. A *P*<0.05 indicates that a difference has statistical significance.

## Results

### Analysis of degree of satisfaction and time to hospital discharge

Sixty one out of 286 cases of patients were very satisfied (21.32%), 201 were satisfied (70.27%), 19 cases were dissatisfied (6.64%), and 5 cases were extremely dissatisfied (1.74%); the total satisfaction rate was 91.61%. There were 72 cases (25.17%) with time to discharge shorter than 1h, 118 cases (41.25%) with a time to discharge of 1 to 3 h, 54 cases (18.88%) with time to discharge of 3 to 6 h, and 42 cases (14.68%) with time to discharge of more than 6 h. The average time to discharge was 1.6±0.8 h. The time to discharge and the satisfaction degree were negatively correlated (r=0.326. *P*=0.028), the longer the time to discharge, the lower the satisfaction degree of patients was.

### Single factor analysis of influence factors

It was analyzed whether individual factors influenced the time to discharge. Gender, payment method and assigned medical department did not have an influence (*P* > 0.05). On the other hand, all other individual factors studied affected the time to discharge. An older patient, a higher income level, an education degree either too low or high, an admission time between 17:00 and 7:59, insufficient understanding of the zoning system and a high score grading the patient’s condition were all important causes leading to a time to discharge ≥2 h. In all these cases the group of patients with a time to discharge longer than two hours differed significantly from the group of patients who had a time to discharge shorter than 2 h (*P*<0.05) (Table 1).

**Table 1:** Single factor analysis of factors influencing the resulting time to discharge

** Factors **	** Case **	** Number of cases with Time to discharge ≥ 2 h **	** χ^2 ^**	***P***
Gender			0.657	0.673
Male	144	22 (15.27)		
Female	142	20 (14.08)		
Age (year old)			4.257	0.039
20∼40 years old	103	8 (7.8)		
40∼65 years old	183	30 (16.4)		
Monthly income (Yuan)			8.372	0.015
<2000	79	5 (6.3)		
2000∼5000	143	22 (15.4)		
>5000	64	15 (23.4)		
Fee payment method			0.267	0.084
Self-paying	121	18 (14.9)		
Health care	165	24 (15.8)		
Education degree			12.288	0.002
Junior school and below	65	14 (21.5)		
High school and polytechnic school	159	13 (8.2)		
Junior college above	62	15 (24.2)		
Treatment department			0.545	0.761
Internal medicine	153	16 (10.5)		
Surgery	83	10 (12.0)		
Other departments	50	4 (8.0)		
Treatment time			14.471	0.001
8:00–16:59	147	11 (7.5)		
17:00–23:59	93	18 (19.4)		
0:00–7:59	46	13 (28.3)		
Understanding of zoning classification			132.402	0.000
Understanding	225	13 (5.8)		
Not understanding	61	44 (72.1)		
Understanding of condition severity score			107.544	0.000
Understood	223	19 (8.5)		
Not understood	63	44 (69.8)		

### Regression analysis results

The above factors were included into a Logistic regression model, the following results were obtained: age income level, education degree, treatment time, understanding of zoning system and condition severity scoring were all independent risk factors leading to a time to discharge ≥ 2 h (*P*<0.05). Meanwhile, gender, fee payment method, and assigned treatment department did not have any effect on the time to discharge (Table 2).

**Table 2:** Regression analysis results

** Factors **	** β **	** Wald **	***P***	** OR **	** 95% CI **
Gender	0.283	1.302	0.623	0.765	−0.626∼2.634
Age	0.316	4.230	0.034	1.964	1.126∼3.629
Monthly income	−0.363	4.521	0.030	1.437	0.535∼2.828
Fee payment method	0.567	1.023	0.074	0.556	−0.526∼1.867
Education degree	0.626	5.627	0.023	1.335	0.326∼4.302
Treatment department	0.213	3.203	0.127	0.632	−0.421∼3.203
Treatment time period	−0.523	7.528	0.008	3.746	1.135∼4.876
Understanding of zoning classification	−0.237	8.302	0.003	3.675	1.546∼5.458
Understanding of condition severity score	−0.074	8.469	0.005	3.025	1.126∼5.635

## Discussion

### Research on emergency

With China’s social and economic developments and a bigger aging population, the individual’s health awareness has been increasing. The disparity between the increasing numbers of health services-seeking individuals and the amount of medical resources available has become a major problem in large general hospitals. When the number of admitted patients exceeds the number of the hospital’s capabilities, an overcrowding phenomenon ensues. The first studies trying to figure out what was going on in these times of overcrowding pointed to sudden increases in the number of medical attention-seekers as the root cause (5). However, more in depth studies have found multiple reasons, and unreasonable retention of the patients in the hospitals is one of the key factors (6).

Overcrowding in the emergency department not only affects the timely assessment and treatment of patients, it also leads to lower satisfaction levels in patients and their relatives, and to increased patient’s suffering. In addition, it brings a sense of defeat to the medical personnel, which affects the efficiency, leads to increased medical error rates, increased numbers of medical disputes, and even higher mortality rates (7). How to solve effectively emergency department overcrowding has become a priority in many hospitals.

### Grading and zoning management model

About 30% of emergency patients are critically ill patients; their illnesses are serious and unpredictable, and carry a high risk of death. They need first diagnosis and timely treatment. In the piloting guidance principles of emergency disease classification and the standardization process of hospital emergency department management issued by China’s Ministry of Health, grading and zoning concepts have been described. The emergency department has been divided into zones 1–4 according to condition severity of the patients and the required medical resources (8). In patients with diseases that may endanger life, or in patients who are not breathing nor have pulse, tracheal intubation should be introduced. In addition, patients with symptoms of acute disturbance of consciousness or coma and patients needing immediate resuscitation should be assigned to zone 1. Zone 2 should include patients with severe pain who score 7 points or more on the condition severity scale (such as those with angina pectoris); patients possibly progressing to zone 1 (disturbance of consciousness, combined injuries); or those whose condition may lead to serious disability; and the critical patients that need arranging for transfer to another facility. Patients without life threatening symptoms and with low occurrence of serious complications should be assigned to zone 3. Finally, the patients without severe symptoms and those judged clinically to require only low level medical attention should be assigned to zone 4.

From the spatial layout, the emergency room should be divided into red, yellow and green zones. The patients of zones 1 and 2 are treated in the red zone. The zone 1 patients should be treated following the immediate treatment principle, and the zone 2 patients following the rapid processing principle. Patients of zone 3 are treated in the yellow zone according to a “call queuing system”. Moreover, patients of zone 4 are to be treated by experienced medical staff in the green zone. Grading and zoning management shall not be stereotyped, and the progress of the condition of patients’ needs to be evaluated periodically in order to update the zoning as needed. Applying the condition grading and zoning management model maximizes the efficacy of resources distribution.

### Factors affecting grading and zoning management in emergency triage

Age, income level, education level, medical-attention-seeking time, understanding of zoning classification, as well as understanding of the condition severity scoring are all independent risk factors leading to a time to discharge greater or smaller than 2 hours, while gender, payment methods, and medical department assignment cannot influence the time to discharge. With an increasing aging population, more elderly patients with higher incidence of severe diseases and complications need emergency department treatment and endure longer times to discharge. The patients with low educational levels, especially those with a level below junior high school have a poor understanding ability and cannot communicate as readily which makes for diagnosis difficulties. On the other hand, those patients with a high level of education may be less willing to allow the treating personnel to guide them because they are weary of the judgment of others and have preconceived ideas of how their treatment should proceed. Patients in the middle-income bracket are not able to pay exclusive medical attention like those in the highest income bracket, but they expect a higher degree of service than those in the lower income bracket who are happy to agree with anything offered to them.

The times to discharge for patients arriving to the emergency department at night are 2 to 4 times longer than those for patients arriving during the day (10). The human resources of the hospitals at night are less than those during the day, and the conditions of the patients at night are often more serious, which explains the longer times to discharge in this case.

Lack of understanding for the zoning system and for the condition severity scoring are important factors affecting the efficiency of emergency triage. When patients do not understand the management system, they are more likely to disagree with their assigned zone and waiting times; they may move around and seek attention repetitively, causing a diversion of resources and longer times to discharge. With the lack of enough understanding for the zoning system, non-critical patients wait to see doctors in the red (more rapid) zone, causing chaos and unreasonable hospital retention times (11–12). An emergency department in disorder creates longer retention times, a diversion of essential resources and an increase in the mortality rate of its patients (13–14).

## Conclusion

Establishing an orderly and effective grading and zoning management model is of great significance for achieving an effective emergency triage. Identification factors leading to unreasonable rates of times to discharge is the first step in order to come up with countermeasures that will improve the efficiency of triage in the emergency departments.

## Ethical considerations

Ethical issues (Including plagiarism, informed consent, misconduct, data fabrication and/or falsification, double publication and/or submission, redundancy, etc.) have been completely observed by the authors.
